# A Novel Microfluidic Platform for Circulating Tumor Cell Identification in Non-Small-Cell Lung Cancer

**DOI:** 10.3390/mi16101136

**Published:** 2025-10-01

**Authors:** Tingting Tian, Shanni Ma, Yan Wang, He Yin, Tiantian Dang, Guangqi Li, Jiaming Li, Weijie Feng, Mei Tian, Jinbo Ma, Zhijun Zhao

**Affiliations:** 1 First Clinical Medical College, Ningxia Medical University, Yinchuan 750001, China; tiantt6130@163.com (T.T.); 13139514013@163.com (S.M.); 230120810451@nxmu.edu.cn (W.F.); mk_runner_high@163.com (J.M.); 2 Department of Internal Oncology, Cancer Hospital, General Hospital of Ningxia Medical University, Yinchuan 750001, China; mgwy1974@163.com; 3 Center of Medical Laboratory, General Hospital of Ningxia Medical University, Yinchuan 750001, China; 18295016264@163.com (H.Y.); D080519D@163.com (T.D.); liguangqi77@163.com (G.L.); lemon6966@163.com (J.L.); 4 School of Medical Laboratory, Ningxia Medical University, Yinchuan 750001, China; tff15202635152@163.com; 5 Central Laboratory, Peking University First Hospital Ningxia Women and Children’s Hospital (Ningxia Hui Autonomous Region Maternal and Child Health Hospital), Yinchuan 750004, China; 6 Third Clinical Medical College, Ningxia Medical University, Yinchuan 750001, China

**Keywords:** non-small-cell lung cancer, circulating tumor cells, microfluidics, acoustic, amplification refractory mutation system

## Abstract

Circulating tumor cells (CTCs) are crucial biomarkers for lung cancer metastasis and recurrence, garnering significant clinical attention. Despite this, efficient and cost-effective detection methods remain scarce. Consequently, there is an urgent demand for the development of highly sensitive CTC detection technologies to enhance lung cancer diagnosis and treatment. This study utilized microspheres and A549 cells to model CTCs, assessing the impact of acoustic field forces on cell viability and proliferation and confirming capture efficiency. Subsequently, CTCs from the peripheral blood of patients with lung cancer were captured and identified using fluorescence in situ hybridization, and the results were compared to the immunomagnetic bead method to evaluate the differences between the techniques. Finally, epidermal growth factor receptor (EGFR) mutation analysis was conducted on CTC-positive samples. The findings showed that acoustic microfluidic technology effectively captures microspheres, A549 cells, and CTCs without compromising cell viability or proliferation. Moreover, EGFR mutation analysis successfully identified mutation types in four samples, establishing a basis for personalized targeted therapy. In conclusion, acoustic microfluidic technology preserves cell viability while efficiently capturing CTCs. When integrated with EGFR mutation analysis, it provides robust support for the precise diagnosis and treatment of lung cancer as well as personalized drug therapy.

## 1. Introduction

Lung cancer is the foremost cause of cancer-related mortality globally, presenting a significant threat to human health [1]. The majority of patients receive a diagnosis at an advanced stage, resulting in a five-year survival rate of merely 5% [2]. Early detection and timely intervention in tumor cases substantially enhance patient survival rates. Consequently, early diagnosis and treatment are crucial for improving prognosis and extending patient lifespan.

Histopathological examination remains the “gold standard” for lung cancer diagnosis, permitting direct microscopic analysis of pathological tissues to determine tumor type and stage accurately, thus guiding clinical decisions [3,4]. Despite its accuracy, this invasive procedure carries risks such as pain, bleeding, and infection. Challenges are amplified in advanced lung cancer, where tumor puncture is particularly risky and obtaining adequate samples is difficult. Conversely, liquid biopsy offers a non-invasive alternative, minimizing patient harm and enabling repeated sampling for real-time tumor monitoring. It effectively captures the spatial heterogeneity and evolution of tumors, addressing the limitations of tissue biopsy, which only reveals localized characteristics [5].

Circulating tumor cells (CTCs), a key marker for liquid biopsies, have recently demonstrated significant promise in diagnosing and prognosticating lung cancer [6]. CTCs are tumor cells that detach from primary or metastatic sites and enter the bloodstream, potentially spreading to tissues or organs, such as lymph nodes, the liver, kidneys, the brain, and bones [7,8,9,10]. CTCs contain abundant biological information and can provide various types of molecular dynamic information, including DNA, RNA, and proteins, offering a unique perspective for in-depth analysis of the biological characteristics of tumor cells [11]. Leveraging this advantage, CTC analysis facilitates real-time monitoring of tumor progression and precise evaluation of treatment efficacy, thereby laying a critical foundation for personalized and timely treatment modifications. Though CTC detection provides benefits, such as minimal invasiveness, high specificity, and the ability to reflect tumor heterogeneity [12], clinical application remains challenging due to the extremely low concentration of CTCs (1–10 cells/mL) in blood and their inherent heterogeneity [13].

The isolation and capture of CTCs depend on their distinct physical attributes, such as size, deformability, dielectric properties, and hydrodynamics, as well as their biological markers, including epithelial cell adhesion molecules [14]. Recently, integrating various physical field technologies—acoustic, optical, electrical, and magnetic—into microfluidic systems has significantly enhanced the purity and efficiency of CTC sorting [15]. Acoustic fields enable contactless cell manipulation through precise acoustic radiation forces, thereby preserving cell viability [16]. Optical fields utilize optical tweezers to capture and precisely arrange individual cells [17]. Electrical fields utilize dielectrophoretic forces for high-throughput sorting based on cellular electrical properties, facilitating downstream molecular analysis while maintaining cell integrity [18]. Magnetic fields use functionalized magnetic beads to specifically identify and separate target CTCs [19]. Among these technologies, acoustic field technology is distinguished by its unique advantages. It non-invasively manipulates cells using a non-contact acoustic radiation force, preserving their intrinsic state and viability. This method offers convenient operation, high efficiency, and controllability. While bioaffinity methods are also widely employed, their limitations, such as the omission of epithelial–mesenchymal transition-type CTCs, are increasingly recognized. To address this, research is trending towards multimodal sorting strategies, like combining acoustic fields with microfluidic chips. By applying an acoustic field force within these chips, cell capture is enhanced [20], providing a more precise sample basis for lung cancer gene research.

Research indicates that lung cancer occurrence and progression are closely linked to multiple gene mutations [21]. Among these, mutations in the epidermal growth factor receptor (EGFR) gene are a key driver of non-small-cell lung cancer (NSCLC) pathogenesis and progression [22]. The amplification refractory mutation system polymerase chain reaction (ARMS-PCR) employs a unique primer design, offering high sensitivity and specificity in detecting gene mutations. This method has become a leading technology in personalized tumor molecular diagnostics [23]. Utilizing ARMS-PCR for detecting EGFR mutations in patients with lung cancer enables rapid and precise identification of specific EGFR mutation types [24,25].

This study integrates acoustic microfluidic technology with ARMS-PCR to develop a comprehensive platform for lung cancer diagnosis and treatment, advancing precision medicine. Utilizing acoustic field forces, the platform effectively captures microspheres, A549 cells, and CTCs within a microfluidic chip, ensuring cell viability and preserving biological characteristics. By incorporating ARMS-PCR, it detects EGFR gene mutations in captured CTCs, providing crucial molecular data for personalized treatment. This platform not only supports lung cancer diagnosis, treatment guidance, and efficacy assessment but also opens new avenues for clinical research and practice.

## 2. Materials and Methods

### 2.1. Specifications of Microfluidic Chips

The microfluidic chip used in this experiment has external dimensions ranging from 12 × 11 mm to 30 × 20 mm, with a thickness of 0.5 to 1 mm. The inlets and outlets have a diameter of 1 mm and feature through-holes. The flow channel measures 10 × 1 mm in a serpentine configuration, with a depth of 0.2 mm. Micropores have a diameter of 0.1 mm, spaced 0.2 mm apart center to center, and a depth of 80 µm. The glass coverslip has external dimensions of 50 × 40 mm and a thickness of 0.15 mm.

### 2.2. Construction of Microfluidic Instruments

The microfluidic chip features circular micropores and is constructed from transparent polydimethylsiloxane (PDMS) material. A piezoelectric ceramic sheet, functioning as a sensor, converts electrical energy into mechanical vibrations and is the component most intimately linked to the microfluidic chip within the sorting system. The disc-shaped piezoelectric ceramic transducer used in this study has a diameter of 20 ± 0.5 mm and a total thickness of 0.42 ± 0.03 mm, composed of FT-27T-4.0A1 hard piezoelectric material. Following the bonding of the PDMS chip to a glass coverslip, a piezoelectric ceramic plate is attached to the underside, and thin tubes are inserted at both ends to link the sample injection device. The chip is then positioned on a microscope, with wires connecting it to the amplifier, signal generator, and piezoelectric ceramic plate. The frequency and voltage are adjusted, and observations are made using a microscope (OLYMPUS-IX53/Japan/Tokyo/Olympus Corporation).

### 2.3. Experiment on Simulating Cells with Polystyrene Microspheres

To prepare mixed solutions of 8, 15, and 25 μm microspheres with phosphate-buffered saline (PBS), combine 970 μL of PBS with 30 μL of the microsphere solution, ensuring thorough mixing. If adhesion occurs, incorporate 10–30 μL of Tween 80 and mix again. Once the device is connected, rinse the chip with 75% ethanol and use PBS to remove air bubbles. Load the sample, then observe microsphere capture under a microscope. Adjust the voltage, flow rate, and frequency as needed, and document the data.

### 2.4. Study on the Capture Efficiency of A549 Cells by Acoustic Microfluidic Technology

Cultured A549 cells were prepared into suspensions with concentrations of 1 × 10^6^, 1 × 10^5^, 1 × 10^4^, and 1 × 10^3^ cells/mL and then loaded onto the machine. Cell capture was observed microscopically, and the capture efficiency was determined by recording the ratio of captured cells to those passing through the chip per unit time.

### 2.5. Labeling A549 Cells and Investigating Their Capture Utilizing an Acoustic Microfluidic Chip

A549 cells were stained and labeled with the Cell Plasma Membrane Staining Kit with DiI (Red Fluorescence) (DiI) following the manufacturer’s instructions. The experiment consisted of three groups: a. PBS buffer + A549 cell group (10:1 ratio); b. PBS buffer + supernatant blood + A549 cell group, where whole blood from healthy individuals was allowed to stand at room temperature for 10 min and then centrifuged at 12,000 rpm for 5 min, collected supernatant blood, and the above A549 cells suspension, supernatant blood, and PBS buffer were mixed in a 1:1:8 ratio; c. PBS buffer + whole blood + A549 cell group, where the A549 cells suspension, whole blood, and PBS buffer were mixed in a ratio of 1:1:8. After being passed through the microfluidic chip, cell capture was observed across all groups. Captured cells from group C underwent Calcein Acetoxymethyl Ester (Calcein AM) viability staining, followed by centrifugation at 100× *g* for 5 min at room temperature, and were washed twice with PBS. The Calcein AM assay working solution was added, and cells were incubated at 37 °C in the dark for 30 min. Staining was observed using a fluorescence microscope (OLYMPUS-IX53/Japan).

### 2.6. Capture of CTCs from the Peripheral Blood of Patients with Lung Cancer

#### 2.6.1. Inclusion and Exclusion Criteria for Patients with Lung Cancer

Inclusion criteria: a. diagnosed with NSCLC via clinical or pathological assessment; b. aged 20 to 80 years, any gender; c. predominantly at an advanced stage (Stage III/IV) with tumor metastasis. Exclusion criteria: a. presence of severe hematological disorders; b. diagnosis of multiple malignant tumors; c. presence of severe immune dysfunction. Informed consent from all participants were obtained in the study. The study was conducted in accordance with the Declaration of Helsinki, and the protocol was approved by the General Hospital of Ningxia Medical University Medical Research Ethics Review Committee (KYLL-2022-0782) on 27 October 2022.

#### 2.6.2. Collection of Peripheral Blood from Patients with Lung Cancer for CTC Capture by Acoustic Microfluidic Technique

Five milliliters of peripheral blood were collected from patients with lung cancer. After lysing erythrocytes with RBC lysate, ultrasound was employed for acoustic microfluidic capture. The captured cells were stained, dried overnight as instructed, and then subjected to immunostaining and fluorescence in situ hybridization (iFISH). Finally, fluorescence microscopy (Nikon/Japan/Tokyo) was used to photograph the slides to determine whether the captured cells were CTCs.

#### 2.6.3. Peripheral Blood Was Collected from the Same Patient, and CTCs Were Detected Using Microfluidics Combined with Immunomagnetic Bead Technology, and Comparison of the Differences Between the Two Methods 

Execute the protocol by sequentially performing cell separation, incubation, magnetic bead separation, and staining on a 5 mL sample of peripheral blood from the same patient. After staining, examine the final slide under a microscope (AXIO Imager Z2, Zeiss/Germany/Oberkochen) to determine if the cells isolated using microfluidics with immunomagnetic beads are CTCs. Compare these findings with CTCs captured via acoustic microfluidic technology.

### 2.7. Detection of EGFR Genetic Mutations in Patients with Lung Cancer Using the ARMS-PCR Method

Collect 10 mL of peripheral blood from patients whose CTCs have been identified through the above operations. Obtain 10 mL of peripheral blood from patients with identified CTCs. Centrifuge the sample at 2000× *g* for 10 min, and collect the supernatant, which constitutes the serum. Follow the provided protocol to extract DNA and determine its concentration. Set up the ARMS-PCR reaction system according to the guidelines, conduct on-machine detection, and analyze the results.

### 2.8. Data Analysis

Data analysis was performed using SPSS 26, with results expressed as mean ± standard deviation. Each test was conducted in triplicate, and statistical significance was set at *p* < 0.05. Graphical analysis was executed using GraphPad Prism 9.5.0. Fluorescence staining quantification was performed using ImageJ 8, and image composition was completed with Adobe Illustrator 2022.

## 3. Results

### 3.1. Model of Acoustic Microfluidic Chip and Mechanism of Capture

Acoustic microfluidic technology integrates acoustic waves with microfluidics, utilizing acoustic radiation and Stokes drag forces from acoustic streaming to manipulate the movement of particles and cells for efficient and rapid cell separation and capture [26,27,28]. Studies indicate that these forces are closely linked to cell diameter; larger cells experience a stronger capture force under identical acoustic conditions than smaller cells [26]. By modifying parameters like voltage and frequency, the micropores in the acoustic microfluidic chip can selectively capture target cells, which is central to its capture mechanism.

**Figure 1:**
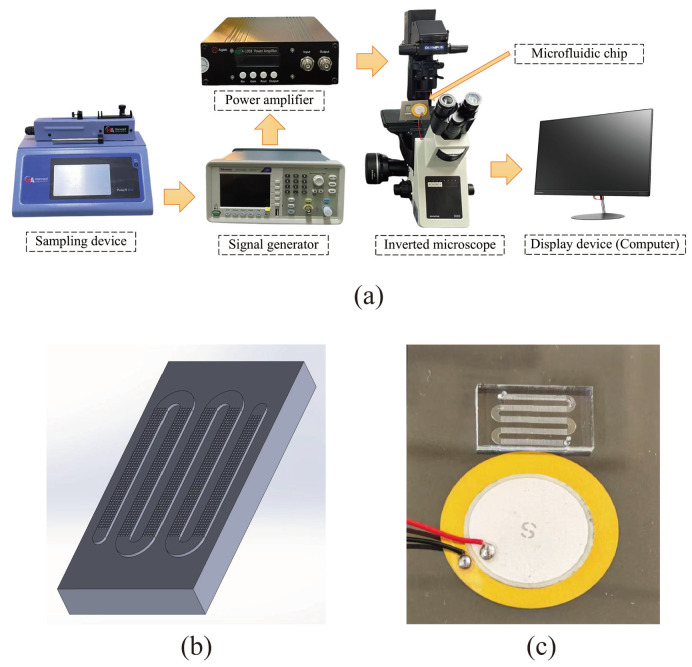
Diagram of materials for building acoustic microfluidic technology. (**a**) Diagram of the acoustic microfluidic device; (**b**) microfluidic chip model diagram; (**c**) microfluidic capture chip actual picture.

**Figure 2:**
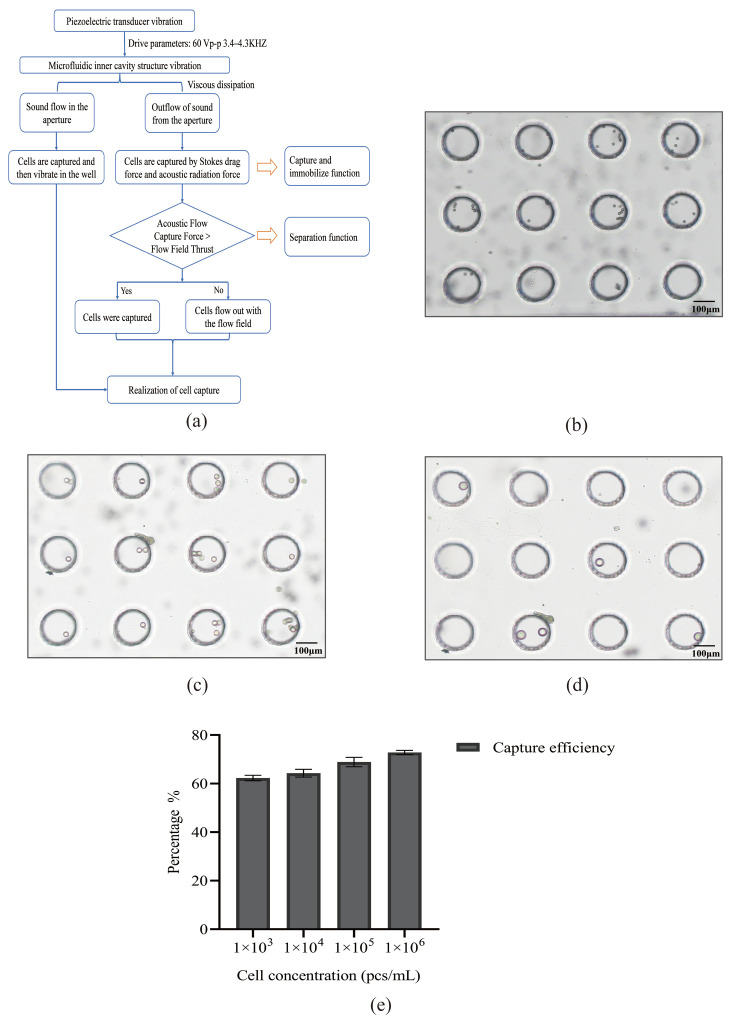
Schematic diagram of acoustic microfluidic technology for capture, and a schematic illustration of microsphere capture. (**a**) Overview of the capture principle of the acoustic microfluidic chip; (**b**) microscope image of an 8 μm microsphere captured by the chip; (**c**) microscope image of a 15 μm microsphere captured by the chip; (**d**) microscope image of a 25 μm microsphere captured by the chip; (**e**) graph of the capture efficiency of A549 cells under different concentration gradients.

The acoustic microfluidic device is depicted in Figure 1a, with its chip model and actual chip illustrated in Figure 1b and 1c, respectively. Upon activating the device, the device is adjusted to a specific voltage and frequency. The application of an acoustic field force amplifies the sine wave from the signal generator, causing the piezoelectric transducer to vibrate. This vibration is transmitted to the microporous array structure within the PDMS chip. Viscous dissipation generates a pressure differential around the micropores, creating acoustic flow inside and outside the pores. Figure 2a presents the schematic of cell capture. A micro-syringe pump injects the detection sample into the chip. Larger cells experience an acoustic flow capture force exceeding the thrust of the flow field, resulting in their capture, while smaller cells continue with the flow field. This entire process is observable in real time through an inverted microscope. By placing the chip on the microscope stage, captured cells in the micropores are visible in real time by adjusting the lens and focus knob.

Upon application of an acoustic field, the liquid advances under the influence of the acoustic force. Captured cells jump within the micropores, while uncaptured cells are carried away by the flow. The captured cells remain stable in the micropores during the acoustic field’s action, enabling real-time observation or subsequent procedures. Once the acoustic field is deactivated, these cells resume flowing, allowing for cell sorting. By modifying parameter settings, cells of varying diameters and those in different solute conditions (such as PBS or whole blood) can be effectively captured.

### 3.2. Experiment on Simulating Cells with Polystyrene Microspheres by Using Acoustic Microfluidic Technology

In the acoustic microfluidic device, 8 μm polystyrene microspheres were trapped in the microchannels and exhibited a jumping motion at 2.5 V, 2 μL/min, and 3.5 kHz (Figure 2b). Adjusting the parameters to 3 V, 3 μL/min, and a frequency of 3.9–4.1 kHz resulted in the capture and similar jumping behavior of both 15 μm and 25 μm microspheres (Figure 2c,d).

### 3.3. Study on the Cell Capture Efficiency of Lung Cancer A549 Cells by Acoustic Microfluidic Technology

Under conditions of 3 V, 3 μL/min, and 4.0 kHz, experiments with varying concentrations of A549 cells demonstrated that capture efficiency increased significantly with higher concentrations, stabilizing above 70% at elevated levels (Figure 2e). This suggests that increasing the initial cell count is essential for optimizing CTC capture efficiency.

### 3.4. Investigation of the Capture of Pre-Labeled A549 Cells Using Acoustic Microfluidic Technology

**Figure 3:**
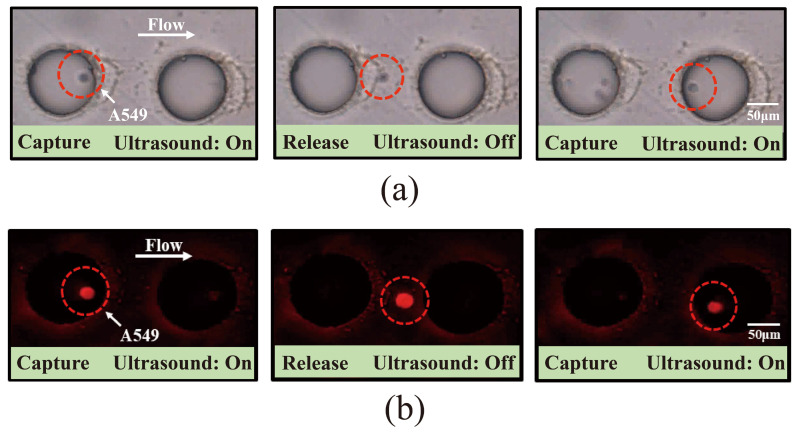
Acoustic microfluidic capture of pre-labeled A549 cells. (**a**) A549 cells in PBS captured by the acoustic microfluidic chip under bright-field microscopy; (**b**) A549 cells in PBS captured by the acoustic microfluidic chip under fluorescence microscopy.

**Figure 4:**
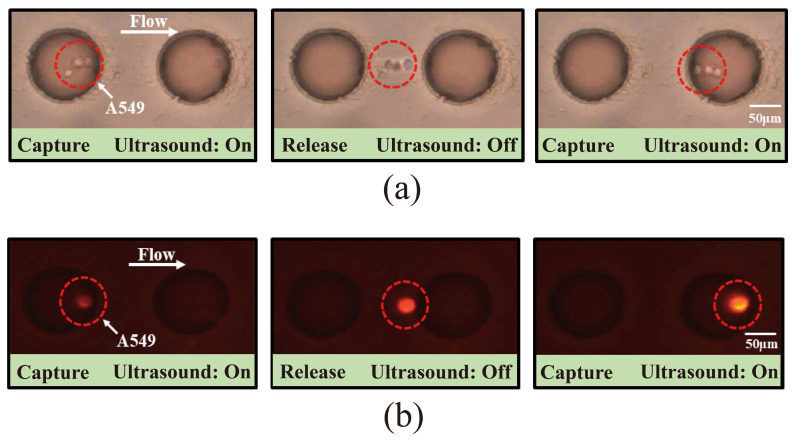
Acoustic microfluidic capture of pre-labeled A549 cells. (**a**) Bright-field image showing A549 cells captured by the acoustic microfluidic chip after mixing with the peripheral centrifuged supernatant blood of a healthy individual; (**b**) fluorescence image showing A549 cells captured by the acoustic microfluidic chip after mixing with the peripheral centrifuged supernatant blood of a healthy individual.

DiI-labeled A549 cells were successfully captured in PBS at an applied voltage of 3 V, a flow rate of 0.6 μL/min, and a frequency of 3.2 kHz. Microscopic observation revealed the captured cells exhibiting beating behavior under bright-field illumination (Figure 3a) and fluorescent labeling (Figure 3b), as shown in a video in Supplementary Materials S1. Reducing the flow rate to 0.5 μL/min enabled the capture of DiI-labeled A549 cells in the supernatant of healthy human peripheral blood samples after centrifugation, with the captured cells visible under both bright-field and fluorescence microscopy and in a video (Figure 4a,b, Supplementary Materials S2). These results demonstrate the efficient capture of labeled A549 cells by the acoustic field, both in PBS and in the supernatant of human blood samples.

**Figure 5:**
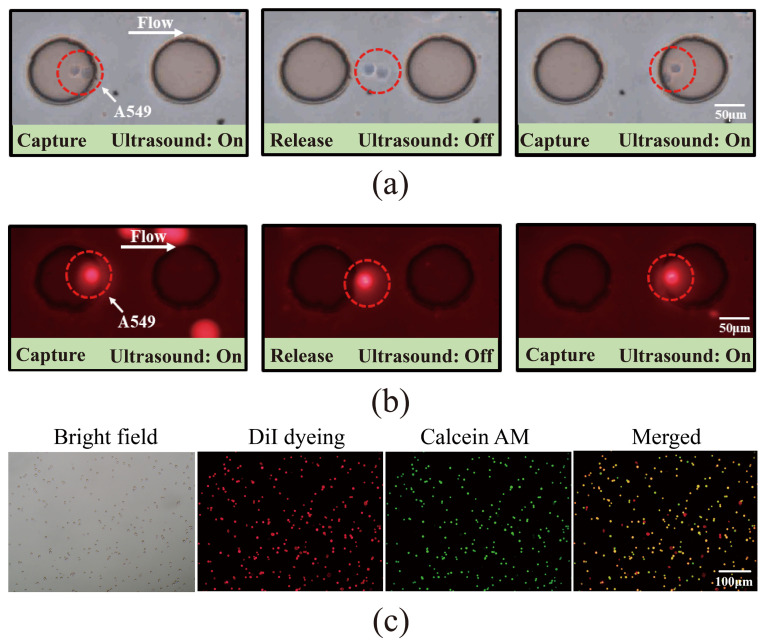
Acoustic microfluidic capture of pre-labeled A549 cells. (**a**) Bright-field view of A549 cell capture on an acoustic microfluidic chip following admixture with healthy human peripheral blood; (**b**) fluorescence view of A549 cell capture on an acoustic microfluidic chip under the same conditions; (**c**) cell viability staining of A549 cells after being captured in healthy human peripheral blood.

To assess the device’s capture performance in complex environments, DiI-labeled A549 cells were introduced into whole blood samples. Successful cell capture was achieved at 3 V, 0.3 μL/min, and 3.2 kHz, as shown in the bright-field imaging (Figure 5a). However, due to the complexity of whole blood, cell types were indistinguishable in bright field alone. Fluorescence imaging confirmed the red cells as target A549 cells (Figure 5b), with a video provided in Supplementary Materials S3. Calcein-AM staining further differentiated cell types: live A549 cells were positive in both bright field and double fluorescence (red + green), dead A549 cells showed red fluorescence but not green, live leukocytes were positive in green fluorescence only, and dead leukocytes were visible only in bright field (Figure 5c). Table 1 demonstrates how DiI-labeled cells can be distinguished from other blood cells under a microscope based on the staining results after Calcein-AM staining. This experiment demonstrates that acoustic microfluidic technology can capture cells in complex blood samples and differentiate cell viability through staining.

### 3.5. Utilizing Acoustic Microfluidics Technology to Isolate CTCs from Patients with Lung Cancer

Suitable cases were selected based on the specified inclusion and exclusion criteria, and the clinical data for these cases were systematically gathered and organized, as detailed in Table 2.

**Figure 6:**
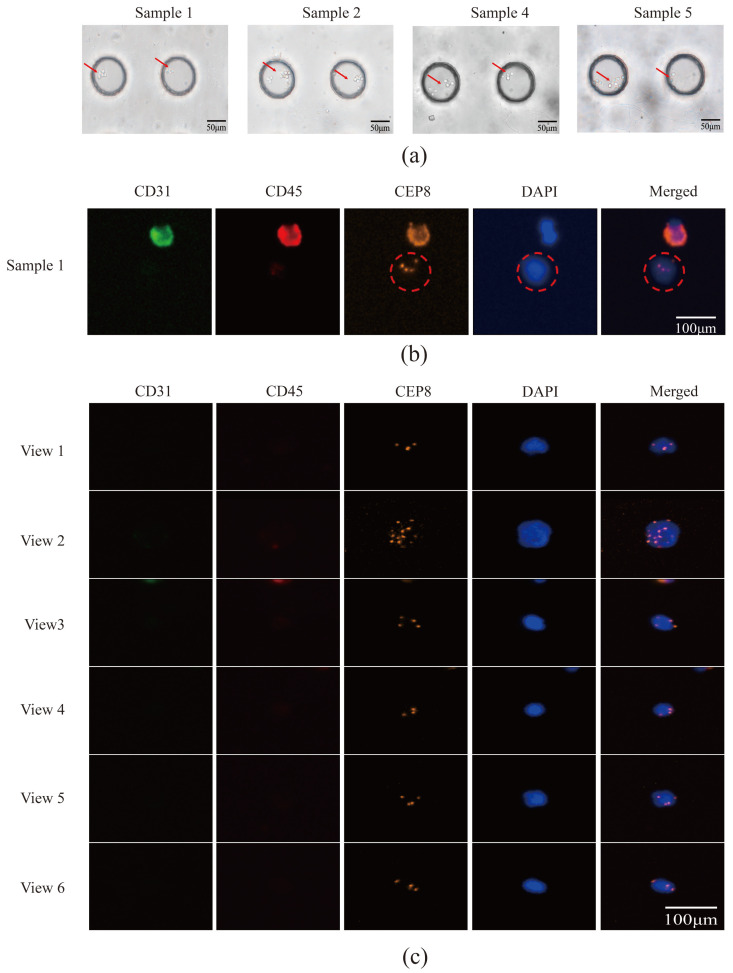
CTCs were captured using acoustic microfluidic technology from the peripheral blood of patients with lung cancer. (**a**) Cell capture of some samples (samples 1, 2, 4, 5) in the acoustic microfluidic device; (**b**) CTCs in sample 1 identified via iFISH fluorescent staining (red circle); (**c**) CTCs in sample 2 identified via iFISH fluorescent staining, the cells in fields of view 1–6 were all CTCs.

**Figure 7:**
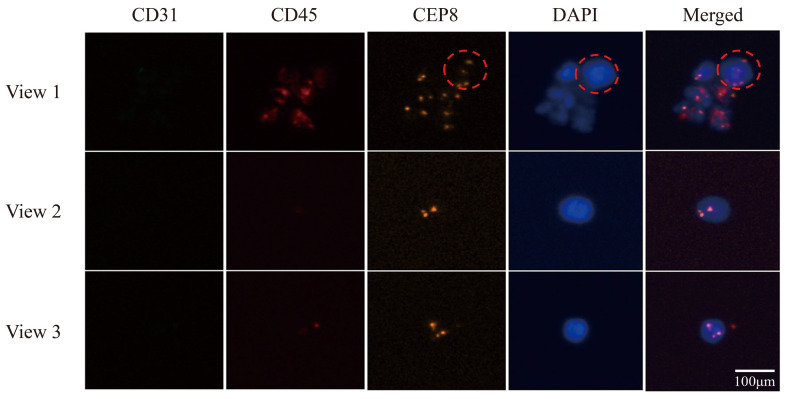
CTCs were captured using acoustic microfluidic technology from the peripheral blood of patients with lung cancer, with fluorescent staining of CTCs identified by iFISH in sample 3, the red circle cells in field of view 1 were CTCs, the cells in fields of view 2 and 3 were all CTCs.

**Figure 8:**
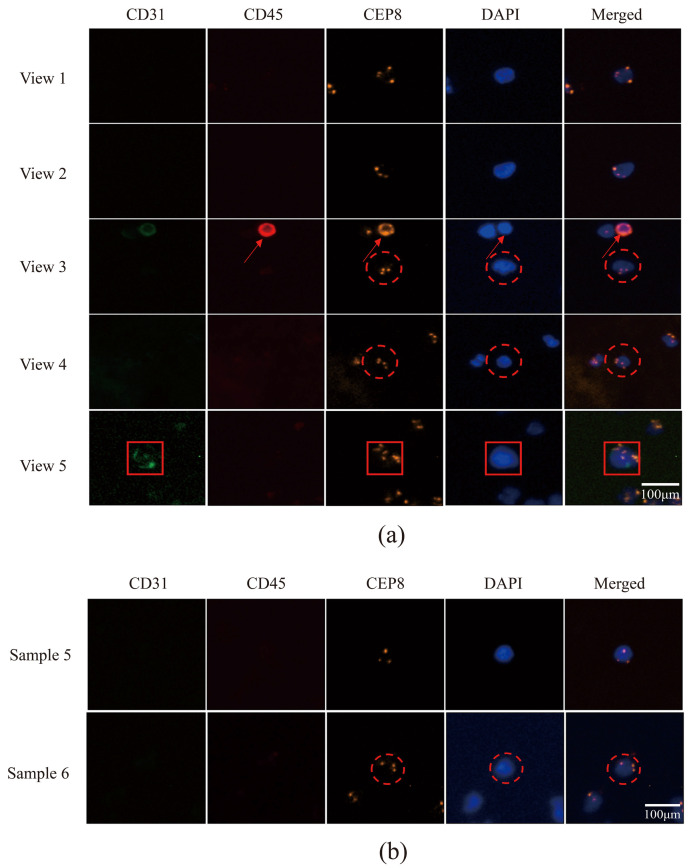
CTCs were captured using acoustic microfluidic technology from the peripheral blood of patients with lung cancer. (**a**) Fluorescent staining of CTCs, leukocytes, and endothelial cells identified by iFISH in sample 4, the cells in fields of view 1 and 2, the red circle cells in fields of view 3 and 4 were CTCs, the cell identified by the red arrow in field of view 3 was a leukocyte. The cells within the red box in field of view 5 were endothelial cells; (**b**) CTCs identified by iFISH fluorescent staining in samples 5 and 6, the cells in sample 5 were CTCs, the cells labeled with red circle in sample 6 were CTCs.

Peripheral blood samples from patients with lung cancer were captured using an acoustic microfluidic device. Subsequent liquid cell staining and iFISH analyses were performed. The acoustic microfluidic chip successfully captured peripheral blood cells from six samples under the conditions of 3 V, 3 μL/min, and 4.0 kHz, as shown in Figure 6a. The captured cells were clearly visible under a microscope. Due to the similar cell sizes of CTCs and white blood cells [29], both cell types may be captured within a similar acoustic field frequency range, rendering morphological observation alone insufficient for distinguishing CTCs from white blood cells. To reliably identify the captured cells, the iFISH detection method was employed for verification. The iFISH analysis revealed the presence of one, six, and three CTCs in samples 1, 2, and 3, respectively (Figure 6b,c; Figure 7), four CTCs in sample 4 (Figure 8a), and one CTC each in samples 5 and 6 (Figure 8b).

CTC identification relies on the expression profiles of three markers: the platelet endothelial cell adhesion molecule (CD31), the leukocyte common antigen (CD45), and 4′,6-diamidino-2-phenylindole (DAPI) [30,31,32,33], alongside Chromosome 8 Centromere Probe (CEP8) labeling. CEP8 is used to label the chromosomal positions of the detected cells. CD31 distinguishes CTCs from circulating tumor vascular endothelial cells, enhancing specificity by excluding endothelial cell contamination, where endothelial cells express CD31. CD45 differentiates leukocytes from CTCs, ensuring the captured cells are not hematopoietic, as leukocytes are CD45-positive. Both CTCs and leukocytes exhibit positive DAPI staining because they have nuclei. Notably, in certain cases (Figure 6b), leukocytes may also express CD31. Figure 6b illustrates the CTCs detected in sample 1, with the CEP8 probe indicating chromosomal positions from iFISH. The marked cells are CTCs, while those above are leukocytes.

Immunofluorescent labeling analysis revealed that all cells in sample 2 were CTCs, characterized by double negativity for CD31 and CD45 and positivity for DAPI expression (Figure 6c). In sample 3, the labeled cells in field of view 1 were CTCs, while the unlabeled cells were CD45-positive leukocytes; the cells in fields of view 2 and 3 were all CTCs (Figure 7). Sample 4 contained CTCs in fields of view 1 and 2, as well as in the cells indicated by the dotted lines in fields of view 3 and 4, while the cell identified by the arrow in field of view 3 was a leukocyte. The cells within the box in field of view 5 were endothelial cells (Figure 8a). The cells in sample 5 were CTCs; the cells labeled in sample 6 were also identified as CTCs (Figure 8b).

### 3.6. CTCs in the Peripheral Blood of Patients with Lung Cancer Were Detected Using a Microfluidic Technique Combined with Immunomagnetic Bead Separation and Compared to Acoustic Microfluidic Technology

**Figure 9:**
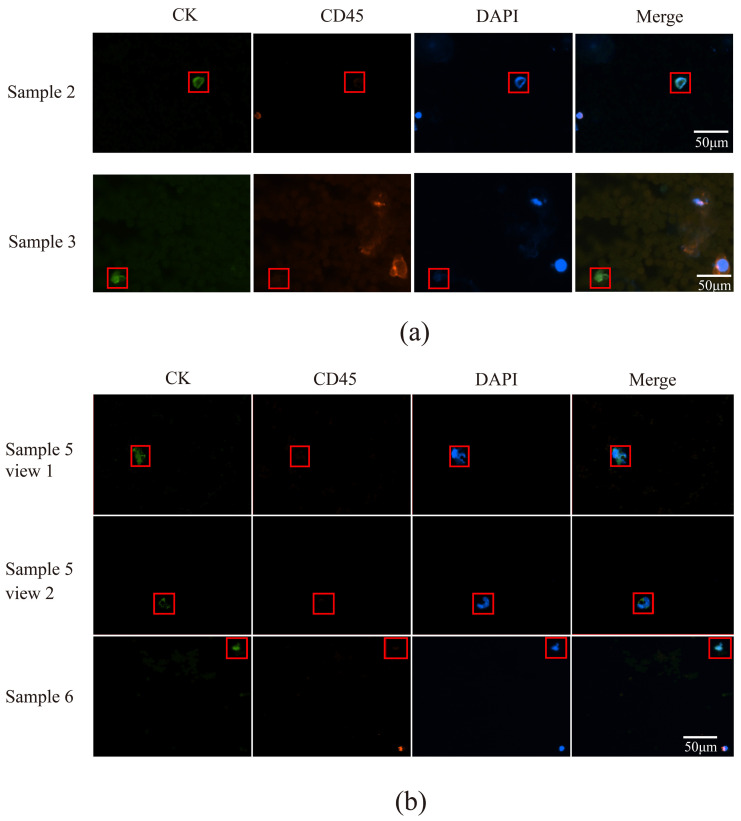
CTC fluorograms obtained using a microfluidic device with immunomagnetic bead separation. (**a**) Fluorescent staining-based CTC maps for samples 2 and 3, the cells with red box were CTCs. (**b**) Fluorescent staining-based CTC maps for samples 5 and 6, the cells with red box were CTCs.

CTCs were identified in all six samples using the microfluidics–immunomagnetic bead separation approach, which employs cytokeratin (CK), CD45, and DAPI as key markers. CK is a specific marker for CTCs, and its positive expression indicates the presence of CTCs [34]. CTCs were defined as CK-positive, CD45-negative, and DAPI-positive cells. The cells observed in samples 2 and 3 in Figure 9a were all identified as CTCs. Figure 9b shows the cell expression patterns in different fields of view of samples 5 and 6, where the cells marked with squares were identified as CTCs, and the unmarked cells were white blood cells. The numbers of CTCs detected in the six samples using the microfluidics–immunomagnetic bead separation and the acoustic microfluidic technologies were statistically analyzed, as presented in Table 3.

### 3.7. Detection of Genetic Mutations in Patients with Lung Cancer Using the ARMS-PCR Method

ARMS-PCR identified EGFR mutations in four out of six patients with lung cancer, as detailed in Table 4. The detected mutations comprised an exon 19 deletion (19-del), a leucine-to-arginine substitution at position 858 (L858R) in exon 21, a serine-to-isoleucine substitution at position 768 (S768I) in exon 20, and a threonine-to-methionine substitution at position 790 (T790M) in exon 20.

## 4. Discussion

Lung cancer, a highly malignant tumor, imposes a profound burden on many families [35]. Conventional diagnostic and therapeutic approaches are costly and frequently ineffective. Recently, CTC detection technology has emerged as a promising avenue for early lung cancer screening and diagnosis. Nevertheless, existing CTC detection methods remain expensive and time-consuming.

This study employs acoustic microfluidic technology, integrating acoustic waves with microfluidics, to efficiently and rapidly separate and capture target cells. Initially, polystyrene microspheres were used due to their uniform size, ease of modification, chemical stability, low cost, and high capture efficiency [36]. The diameter of CTCs typically ranges from 15 μm to 25 μm [37], aligning with the microspheres used. Thus, initial capture experiments simulated cells with these microspheres. Subsequently, acoustic field parameters were optimized for capturing A549 cells. Short-term viability and long-term proliferation experiments confirmed that the acoustic field maintains cell viability and biological integrity during capture, a finding supported by multiple studies [38,39]. Detailed experimental data and results are provided in Supplementary Materials S4.

Fluorescently labeled A549 cells were utilized in subsequent experiments to demonstrate the capacity of acoustic microfluidic technology to reliably and efficiently capture cells across diverse solute environments, including PBS and whole blood. DiI was used for fluorescent labeling to specifically trace A549 cells, enabling clear identification in complex optical backgrounds, such as whole blood, and reducing interference from impurities or other cells, thus enhancing detection specificity and reliability. Additionally, to assess the impact of acoustic forces on cell viability, captured cells in peripheral blood were stained with Calcein AM/Propidium Iodide, allowing observation of the viability of DiI-labeled A549 cells post-capture. The capture selectivity of acoustic microfluidic technology for specific cell lines primarily arises from the interplay between the inherent physical properties of the cells (e.g., size, density, compressibility) and the external field parameters (frequency, voltage, flow rate) [40]. Due to their relatively large diameter (approximately 15–20 μm) and high acoustic impedance coefficient, A549 cells are readily captured by the acoustic radiation force under specific acoustic field conditions. In contrast, blood cells (such as red blood cells and lymphocytes) have smaller sizes or markedly different mechanical properties, rendering them less affected by the acoustic field under the same conditions, with slower velocities and, consequently, lower capture efficiency or even an inability to be captured [41,42]. This aligns with existing studies on acoustic microfluidic sorting, demonstrating that optimizing acoustic field conditions, such as frequency and voltage, can achieve efficient and selective cell capture [26].

Differences in CTC detection arise between microfluidics–immunomagnetic bead separation and acoustic microfluidic technologies, with the former identifying more CTCs. This discrepancy may be due to the acoustic microfluidic technology being in the research phase, lacking optimized acoustic field parameters, and exhibiting uneven acoustic force distribution in the capture area. Furthermore, these technologies vary in cell capture, staining, and identification criteria. Acoustic microfluidics employs CD31 and CD45 markers, whereas microfluidics–immunomagnetic bead separation uses CK and CD45. This approach may miss CTCs that have undergone epithelial-to-mesenchymal transition and do not express CK, reducing specificity and possibly detecting non-CTCs.

An acoustic microfluidic chip has enabled the capture of CTCs from the peripheral blood of patients with lung cancer. Unlike the CTC capture technique employing microfluidics combined with the immunomagnetic bead separation method, the acoustic microfluidic approach offers distinct advantages, including the elimination of antibody labeling [43], simplified and cost-effective operation, and the preservation of cell viability. Nonetheless, the microfluidics with immunomagnetic bead separation technique has shown high sensitivity and specificity in detecting various tumor cells and has been successfully applied in clinical CTC studies.

While the acoustic microfluidic technology employed in this study effectively captures CTCs, it detects fewer CTCs than microfluidics combined with the immunomagnetic bead separation method. Enhancing CTC detection sensitivity in the future may involve optimizing chip capture parameters, such as acoustic wave frequency, sound pressure intensity, and microfluidic channel design. Overall, the acoustic microfluidic technology efficiently completed CTC capture within approximately 2 h. When coupled with iFISH, the detection process can be completed within 48 h at a cost of only around CNY 1000, offering significant economic advantages over existing technologies (CNY 3000–4000) and demonstrating strong potential for clinical application.

In the first step of this study, CTCs from patients with lung cancer were captured using acoustic microfluidic technology. Patients with identified CTCs underwent EGFR gene mutation analysis. Personalized clinical treatment strategies were then developed based on the specific mutations detected. The ARMS–PCR technology, approved by the China National Medical Products Administration, is employed for gene mutation detection due to its simplicity, rapidity, and reliability. It is effective in identifying gene mutations at early tumor stages [44]. Details on the mutation sites and structural characteristics of EGFR gene mutations are provided in Figure S3 of Supplementary Materials S4. This study involved the extraction of cell-free DNA (cfDNA) from the peripheral blood plasma of patients with lung cancer for mutation analysis. CfDNA consists of non-cell-bound DNA fragments present in the bloodstream, primarily originating from DNA released during cell apoptosis or necrosis [45]. In patients with cancer, cfDNA includes circulating tumor DNA from tumor cells, which harbors tumor-specific genetic variations, such as insertion/deletion mutations, point mutations, and copy number variations [46]. The detection of EGFR gene mutations typically relies on liquid biopsy technology. This approach involves several key steps. First, the patient’s peripheral blood sample is collected, which contains a variety of normal blood cells as well as, potentially, CTCs that have originated from the tumor and entered the bloodstream. Upon apoptosis or rupture of these CTCs, they release cfDNA into the blood. The blood sample is then centrifuged to isolate the serum, from which the DNA is extracted. Finally, a PCR-based technique, such as ARMS–PCR, is employed to detect the presence of EGFR gene mutations within the cfDNA. This information can then be utilized for tumor diagnosis, selection of targeted therapies, and monitoring of treatment effectiveness.

The 19-del and L858R point mutations are the predominant EGFR gene alterations in patients with NSCLC [47]. From a clinical perspective, patients with NSCLC harboring these two mutations have demonstrated favorable therapeutic responses to epidermal growth factor receptor tyrosine kinase inhibitors (EGFR-TKIs) [48,49], establishing them as an important first-line targeted treatment indication.

Notably, the T790M acquired resistance mutation was identified in the assay [50,51,52]. This mutation commonly emerges after 6–13 months of EGFR-TKI treatment and is a primary mechanism of resistance to EGFR-TKI therapy [53,54,55]. In response, third-generation EGFR-TKIs, such as osimertinib, have been developed that can effectively overcome T790M-mediated resistance [52,56]. Additionally, a relatively rare S768I mutation was detected [57], with an incidence of approximately 0.5–1% in patients with NSCLC. Due to the limited number of cases, the clinical characteristics, prognostic significance, and therapeutic response to EGFR-TKIs of this mutation type remain to be fully elucidated, necessitating further expansion of the sample size for in-depth investigation.

In this study, samples 1, 2, 4, and 6 were positive for EGFR gene mutations; however, due to financial or clinical constraints, these patients did not undergo routine genetic testing. Patient 2 received radiotherapy, while patients 1, 4, and 6 were treated with an immunotherapy-based chemotherapy regimen consisting of tislelizumab, albumin-bound paclitaxel, and carboplatin. Although this regimen is standard for advanced NSCLC, patients identified with EGFR mutations are often prioritized for EGFR-TKI therapies, such as osimertinib or gefitinib, which offer superior efficacy and survival benefits. This highlights the critical role of genetic testing in precision lung cancer treatment. Promoting genetic testing in clinical practice is essential to enhance treatment precision and improve patient survival outcomes. To enhance the accuracy of lung cancer diagnosis and treatment while maximizing clinical benefits, we advocate for the adoption of acoustic microfluidic technology in CTC detection. This technology is vital for evaluating metastasis and recurrence risks. Patients testing positive for CTCs should subsequently undergo EGFR mutation analysis via cell-free DNA. In metastatic lung cancer cases, the ARMS method yields superior accuracy for gene mutation detection. This sequential testing approach can significantly lower patient costs, improve test precision, boost diagnostic efficiency, and optimize medical resource allocation. Additionally, it provides comprehensive molecular data, offering robust diagnostic evidence to aid clinicians in formulating the most effective treatment strategies.

The detection of EGFR gene mutations using cfDNA still faces certain limitations. A major challenge is that cfDNA samples may contain DNA from various sources in the circulating blood, and the presence of this non-tumor-derived DNA can interfere with the accuracy of mutation detection. While current EGFR mutation detection kits demonstrate high sensitivity, directly isolating circulating tumor DNA from the peripheral blood of patients with lung cancer could further improve the detection accuracy and specificity. Future research should focus on optimizing the application of acoustic microfluidic technology for the capture of CTCs, emphasizing enhanced capture efficiency and cell purity. This approach would yield more reliable ctDNA for gene mutation analysis and provide high-quality samples for subsequent studies. Detecting EGFR gene mutations has become a crucial molecular diagnostic tool in NSCLC. Identifying specific mutations—such as 19-del, L858R, and T790M—enables clinicians to select targeted therapies more effectively. This precision medicine approach enhances treatment efficacy while reducing unnecessary drug exposure and potential side effects, thus advancing personalized lung cancer treatment.

In this study, we developed a comprehensive technology transformation pathway, progressing from basic parameter optimization (microspheres to cell lines) to clinical application (CTC detection to drug guidance), confirming the feasibility of combining acoustic microfluidic technology with EGFR detection. This stepwise detection strategy not only validated the effectiveness of acoustic microfluidics in capturing CTCs but also integrated CTC detection with EGFR gene mutation analysis. This method enables dynamic monitoring of patient conditions, facilitating precise lung cancer diagnosis and treatment, thereby holding significant clinical value for improving patient prognosis.

The integration of acoustic microfluidic technology, because of its non-destructive, efficient, and cost-effective CTC capture, with ARMS-PCR for precise genetic testing, facilitates comprehensive precision diagnosis and treatment of lung cancer—from initial diagnosis through treatment monitoring and personalized solution optimization. Despite current limitations, such as small sample sizes, advancements and the integration of additional technologies are anticipated to enhance early screening and dynamic monitoring levels of lung cancer, ushering in a new era of precision in its diagnosis and treatment.

## 5. Conclusions

The acoustic field force in acoustic microfluidics, as a non-contact force, excels in preserving cell integrity and viability in vitro. This technology enables efficient capture of microspheres and lung cancer A549 cells, as well as CTCs from the peripheral blood of patients with advanced lung cancer. This capability not only provides a more robust foundation for clinical disease evaluation but also offers crucial technical support for the diagnosis and treatment of lung cancer. When integrated with ARMS-PCR technology, the platform precisely detects EGFR gene mutations, guides targeted therapies, and advances precision medicine applications.

## Figures and Tables

**Table 1:** The staining differences between DiI-labeled cells and other blood cells after Calcein-AM staining.

	Living A549 Cells	Dead A549 Cells	Living White Blood Cells	Dead White Blood Cells
Bright-field field of view	yes	yes	yes	yes
Red fluorescence field of view	yes	yes	no	no
Green fluorescence field of view	yes	no	yes	no

**Table 2:** Clinical data collected on patients with lung cancer.

Sample Number	Years	Sex	Histological Type	Metastasis	Tumor Staging	Personal History
Number 1	72	Female	Squamous Carcinoma	Bone	cT4N2M1 IV	No history of smoking
Number 2	74	Male	Adenocarcinoma	Head	cT1cN2M1b IVA	Previous history of smoking
Number 3	62	Male	Adenocarcinoma	None	cT1cN0M0 IA3	Previous history of smoking
Number 4	59	Male	Squamous Carcinoma	None	cT3N3M0 IIIc	No history of smoking
Number 5	74	Male	Squamous Carcinoma	Lymphatic Node	cT4N3M1 IV	No history of smoking
Number 6	64	Male	Squamous Carcinoma	None	cT4N3M1 IV	Previous history of smoking

**Table 3:** Statistics on CTC detections.

	Number of CTCs Captured by Acoustic Microfluidics Combined with iFISH	Number of CTCs Captured by Microfluidics Combined with the Immunomagnetic Bead Separation Technique
Number 1	1	52
Number 2	6	17
Number 3	3	13
Number 4	4	8
Number 5	1	9
Number 6	1	1

**Table 4:** Detection of EGFR gene mutations.

Sample Number	Type of EGFR Gene Mutation
Number 1	19-del, L858R mutation
Number 2	S768I mutation
Number 3	None
Number 4	T790M mutation
Number 5	None
Number 6	L858R mutation

## Data Availability

The data that support the findings of this study are available from the corresponding author upon reasonable request.

## Supplementary Materials

The following supporting information can be downloaded at: https://www.mdpi.com/article/10.3390/mi16101136/s1, Supplementary Materials S1: The capture video of diI-labeled A549 in bright field and fluorescent field (in PBS); Supplementary Materials S2: The capture video of diI-labeled A549 in bright field and fluorescent field (in the supernatant of healthy human peripheral blood samples after centrifugation); Supplementary Materials S3: The capture video of diI-labeled A549 in bright field and fluorescent field (in whole blood samples); Supplementary Materials S4: The research of short-term viability and long-term proliferation experiments and the analyze of EGFR mutation sites.

## Abbreviations

The following abbreviations are used in this manuscript:

CTCs	Circulating Tumor Cells
EGFR	Epidermal Growth Factor Receptor
NSCLC	Non-Small-Cell Lung Cancer
ARMS-PCR	Amplification Refractory Mutation System Polymerase Chain Reaction
PDMS	Polydimethylsiloxane
PBS	Phosphate-buffered Saline
DiI	Cell Plasma Membrane Staining Kit with DiI (Red Fluorescence)
Calcein AM	Calcein Acetoxymethyl Ester
iFISH	Immunostaining and Fluorescence in Situ Hybridization
CD31	Cluster of Differentiation 31
CD45	Cluster of Differentiation 45
DAPI	4′,6-diamidino-2-phenylindole
CEP8	Chromosome 8 Centromere Probe
CK	Cytokeratin
19-del	Exon 19 Deletion Mutation
L858R	Leucine 858 Arginine Point Mutation
S768I	Serine 768 Isoleucine Mutation
T790M	Threonine 790 Methionine Mutation
cfDNA	Circulating Free DNA
EGFR-TKI	Epidermal Growth Factor Receptor Tyrosine Kinase Inhibitor
