# Supplementary material for: A Novel Microfluidic Platform for Circulating Tumor Cell Identification in Non-Small-Cell Lung Cancer
Source: Micromachines (Basel). 2025 Oct 1;16(10):1136. doi: 10.3390/mi16101136 (PMC12566543; doi:10.3390/mi16101136)
Supplement: Supplementary file 1 [file micromachines-16-01136-s001.zip › Supplementary Materials S4 The research of short-term viability and long-term proliferation experiments and the analyze of EGFR mutation sites/supplementary material 4 .pdf]

1. Materials and Methods

1.1. A549 Cells of Lung Cancer to Simulate the "CTC" Experiment

1.1.1. *Short-term Cell Viability Study of A549 Cells Captured by Acoustic Microfluidic Technology*

A549 cells, sourced from ATCC and obtained via Haixing Biotechnology Company, were digested, centrifuged, and resuspended in PBS. They were then divided into three groups: acoustic on, acoustic off, and control, each containing 330 μL. The three groups of cells were respectively subjected to experiments on the instrument. Post-experiment, cells from all groups were collected, centrifuged, and stained with Calcein Acetoxymethyl Ester/Propidium Iodide (Calcein AM/PI). They were incubated at 37 °C for 30 minutes, shielded from light, and analyzed using a fluorescence microscope (OLYMPUS-IX53/Japan). The survival rate was subsequently calculated.

1.1.2. *Study of Long-term Cell Proliferation Capacity of A549 Cells Captured by Acoustic Microfluidic Technology*

The cultured A549 cells were prepared as a cell suspension and divided into three groups. A volume of 330 μL of cell suspension was taken from each group on the machine. At the end of the procedure, 100 μL samples from each group were centrifuged at 1000×g for 5 minutes. The supernatant was discarded, and the cell pellets were resuspended, seeded in six-well plates, and incubated at 37°C with 5% CO_2. The cell growth was monitored in the same field of view at 24, 48, 72, and 96 hours. Following the procedures outlined, centrifuge the three solution groups and seed them into 96-well plates (1×10^3 cells/well), incubating at 37°C. At 0, 24, 48, and 72 hours, add a CCK-8: Cell Counting Kit-8 (tradename)(CCK-8) mixture, incubate for 30 to 90 minutes, and measure the CCK-8 absorbance at 450 nm. Similarly, after centrifugation, seed cells from each group into 12-well plates (5×10^4 cells/well) and incubate at 37°C. At 24, 48, 72, and 96 hours, digest the cells, count viable and dead cells using an automatic cell counter, and calculate the survival rate.

2. Results

2.1. Investigation of Short-term Cell Viability in A549 Lung Cancer Cells Utilizing Acoustic Microfluidic Technology

Under conditions of 3 V, 10 μL/min, and 3.4 kHz, fluorescence staining revealed minimal differences between live (green) and dead (red) cells. The viability of A549 cells across the three groups was comparable, with no significant differences observed (Supplementary Figure 1a). The cell survival rate was calculated using software, as shown in Supplementary Figure 1b. The results indicated that the survival rates of all three groups of A549 cells exceeded 80%, with no significant differences, suggesting that the acoustic field force had a negligible impact on cell viability and demonstrated excellent biocompatibility.

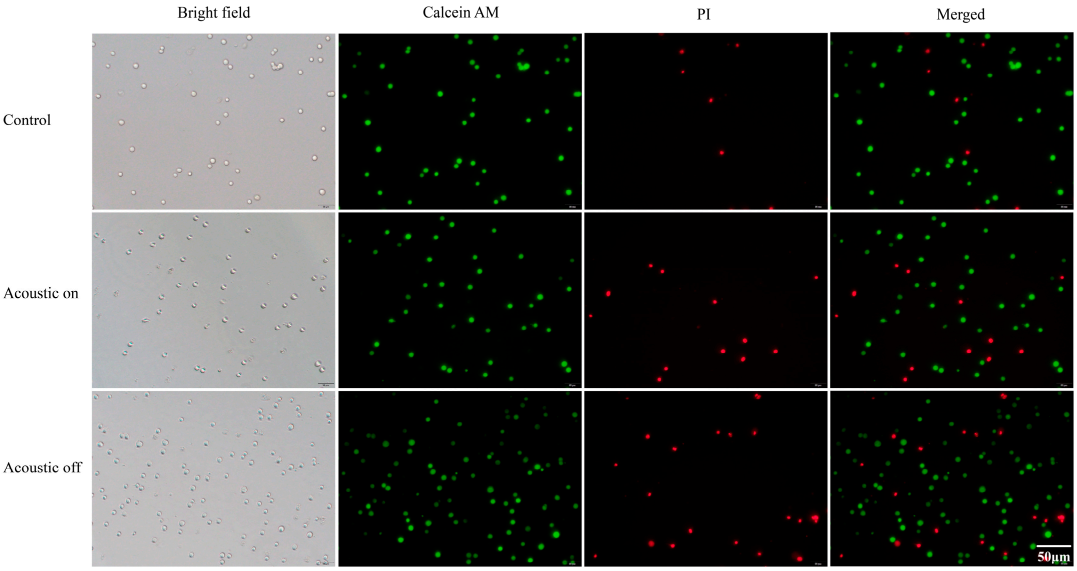

(a)

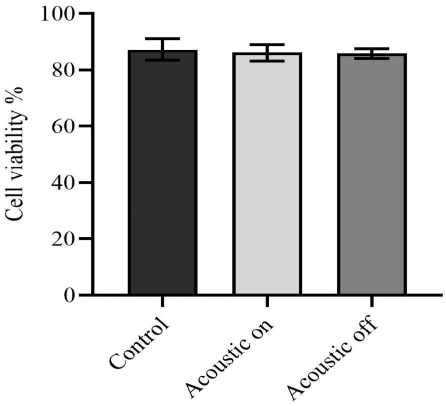

(b)

Supplementary Figure S1. Short-term cell viability study of lung cancer A549 cells captured by acoustic microfluidic technology. (**a**) Short-term cell viability of each group of A549 cells after capture by the acoustic microfluidic chip; (**b**) Histogram of A549 cell viability in each group.

2.2. Long-term Cell Viability Studies of Lung Cancer A549 Cells Captured by Acoustic Microfluidic Technology

Under conditions of 3 V, 10 μL/min, and 3.4 kHz, A549 cells demonstrated growth patterns akin to the control group after 24–96 hours of culture in both acoustic on and off settings (Supplementary Figure 2a). The CCK-8 assay showed an initial decline in survival rates for both groups, likely due to brief air exposure contamination; however, their proliferation capacity recovered to nearly match the control group after culture (Supplementary Figure 2b). Cell counting confirmed that live cell ratios in both acoustic-on and acoustic-off groups were not significantly different from the control group (Supplementary Figure 2c). These findings suggest that acoustic microfluidic technology does not significantly affect cell viability or proliferation capacity.

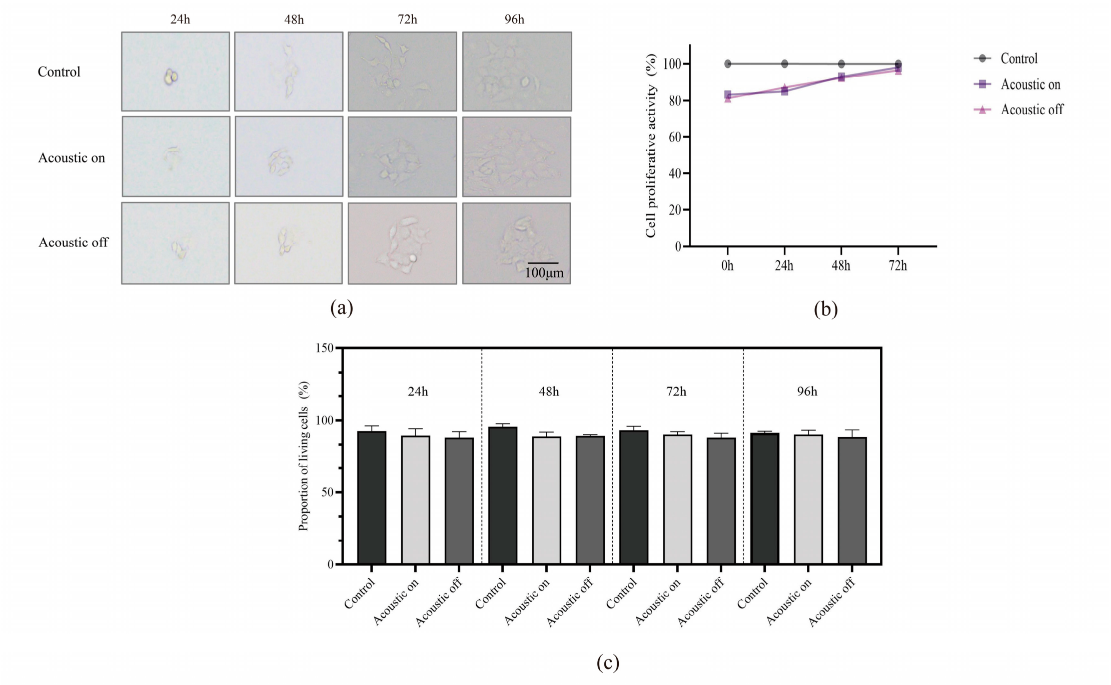

Supplementary Figure S2. Long-term viability assessment of A549 lung cancer cells using acoustic microfluidic technology. (**a**) Growth status graph of A549 cells in the same field of view after 24-96 hours of culture; (**b**) Fold-change graph of cell proliferation based on CCK-8 assay results; (**c**) Histogram of cell counts following 24-96 hours of culture.

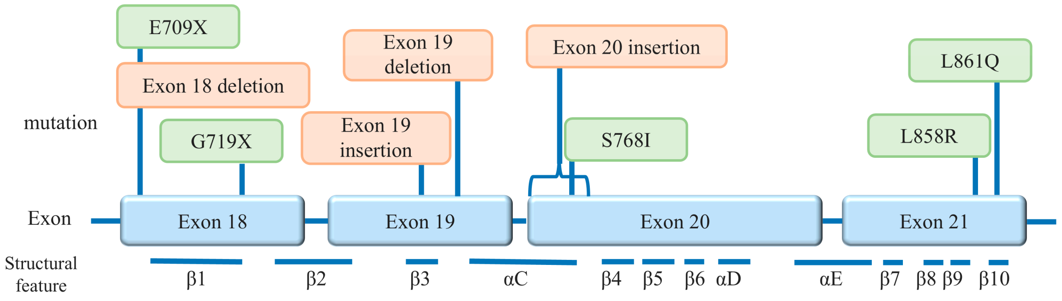

Supplementary Figure S3. Map of the mutation site and structural features of the EGFR gene.
