# Supplementary figures and images for: A Novel Microfluidic Platform for Circulating Tumor Cell Identification in Non-Small-Cell Lung Cancer
Source: Micromachines (Basel). 2025 Oct 1;16(10):1136. doi: 10.3390/mi16101136 (PMC12566543; doi:10.3390/mi16101136)

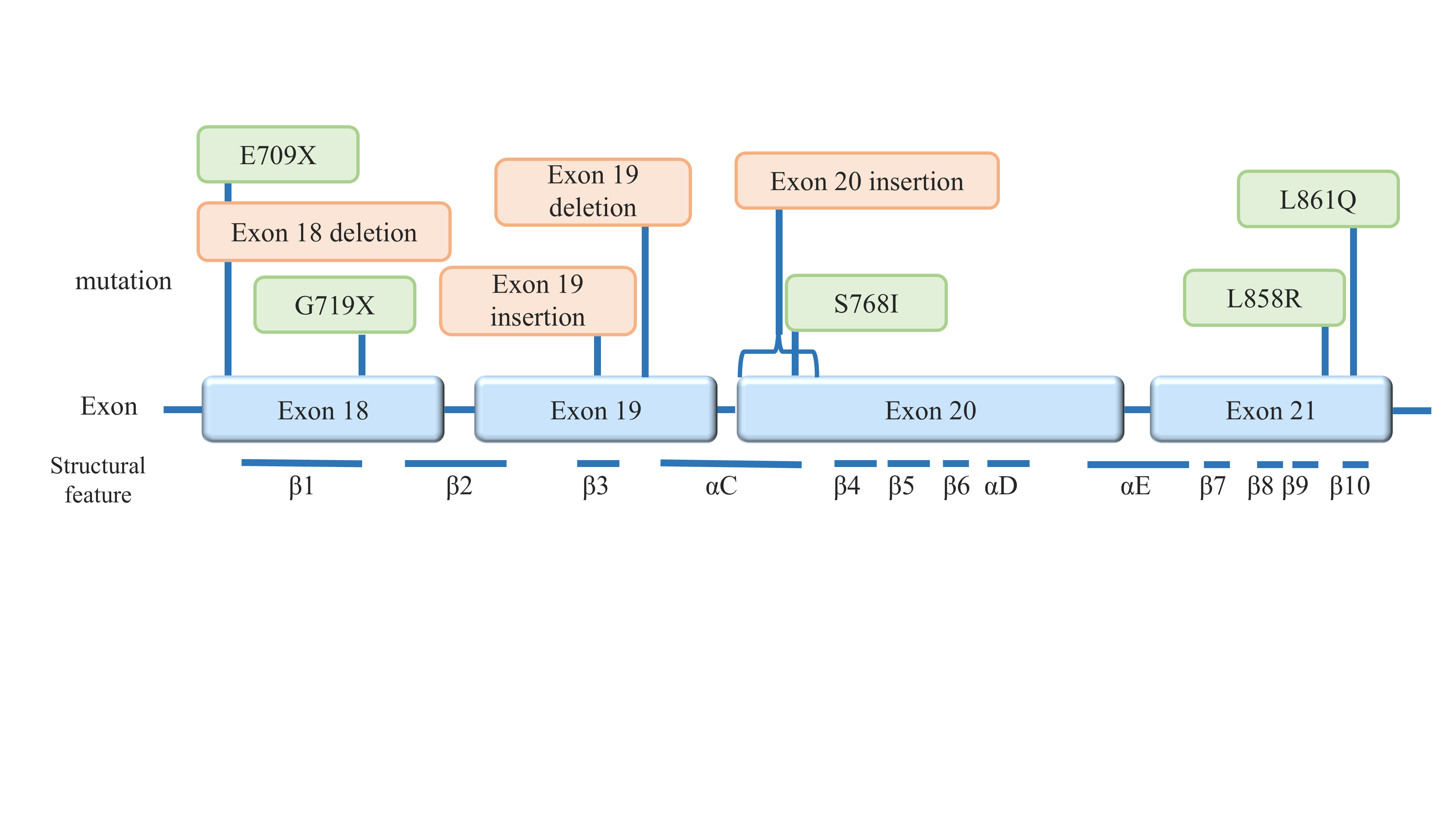

Supplement: Supplementary file 1 [file micromachines-16-01136-s001.zip › Supplementary Materials S4 The research of short-term viability and long-term proliferation experiments and the analyze of EGFR mutation sites/supplementary figure S3.tif]
